# After a Hand Was Lent: Sporadically Experiencing Multisensory Interference During the Rubber Hand Illusion Does Not Shield Against Disembodiment

**DOI:** 10.5334/joc.427

**Published:** 2025-01-17

**Authors:** Julia Eck, David Dignath, Andreas Kalckert, Roland Pfister

**Affiliations:** 1 Department of Psychology III, University of Würzburg, Germany; 2 Department of Psychology, Eberhard Karls University of Tübingen, Germany; 3 Cognitive neuroscience and philosophy, University of Skövde, Sweden; 4 General Psychology, Trier University, Germany; 5 Institute for Cognitive and Affective Neuroscience (ICAN), Trier University, Germany

**Keywords:** Action and perception, Multisensory perception, Learning

## Abstract

Observations from multisensory body illusions indicate that the body representation can be adapted to changing task demands, e.g., it can be expanded to integrate external objects based on current sensorimotor experience (embodiment). While the mechanisms that promote embodiment have been studied extensively in earlier work, the opposite phenomenon of, removing an embodied entity from the body representation (i.e., disembodiment) has received little attention yet. The current study addressed this phenomenon and drew inspiration from the partial reinforcement extinction effect in instrumental learning which suggests that behavior is more resistant to extinction when reinforcement is delivered irregularly. In analogy to this, we investigated whether experiencing occasional visuo-motor mismatches during the induction phase of the moving rubber hand illusion (intermittent condition) would result in slower disembodiment as compared to a regular induction phase where motor and visual signals always match (continuous condition). However, we did not find an effect of reinforcement schedule on disembodiment. Keeping a recently embodied entity in the body schema, therefore, requires constant updating through correlated perceptual and motor signals.

## Introduction

Usually there seems to be no doubt about what and where one’s own hand is, yet visuo-motor illusions show that the cognitive system can be tricked about such seemingly self-evident facts. In one illusion, the rubber hand paradigm, participants see a rubber hand which is arranged in an anatomically plausible position relative to the rest of the body while the corresponding real hand is covered from view. In the passive version of the rubber hand illusion, the experimenter strokes the covered real hand and the corresponding location of the rubber hand for several minutes (Botvinick & Cohen, 1998; Tsakiris & Haggard, 2005). Subsequent to synchronous stroking of the real hand and the rubber hand, participants typically report feeling that the rubber hand is a part of their own body. Such embodiment of an artificial hand can be further induced in an active version of the rubber hand illusion, where movements of the covered real hand trigger movements of the rubber hand due to a mechanical connection between the real and the rubber hand. In the moving rubber hand illusion, for instance, embodiment of the rubber hand emerges from continuous experience of a match between movements of one’s own covered hand and corresponding movements of the rubber hand (Dummer et al., 2009; Kalckert & Ehrsson, 2012, 2014a, 2017). These findings suggest that the illusion might emerge from different combinations of temporally synchronous and spatially compatible cross-modal stimulations (Ehrsson, 2020; Ehrsson et al., 2005; Kalckert & Ehrsson, 2014a).

Embodiment of body-external entities is relevant for a range of real-world applications, including development of prosthetic devices (Castro et al., 2023). Some studies showed that individuals who have lost a limb can experience the rubber hand illusion for their phantom limb if referred phantom limb sensations correspond to visual signals coming from the artificial hand (Ehrsson et al., 2008; Middleton & Ortiz-Catalan, 2020; Page et al., 2018; Rognini et al., 2019; Rosén et al., 2009). These findings suggest that like embodiment of a rubber hand in individuals who still have both of their hands also embodiment of a prosthesis in individuals who have lost a limb possibly emerges from similar mechanisms of integrating cross-modal sensations.

Transferring embodiment research to real-world application requires the experimental effects to last on meaningful timescales. Interestingly, the fate of the rubber hand illusion after its initial onset has only recently been addressed by empirical studies (Abdulkarim et al., 2021; Eck et al., 2022; Eck & Pfister, 2024; Perepelkina et al., 2018; Pfister et al., 2021). These studies observed gradual disembodiment of the artificial hand as soon as the illusion was no longer reinforced by continued embodiment experiences. Moreover, a single mismatch of own sensations and observed stimulation of the rubber hand abolished embodiment immediately (Pfister et al., 2021). This indicates that prosthesis users might start to disembody a previously embodied prosthesis as soon as the experience of corresponding visuo-motor or visuo-tactile sensations is cancelled e.g., due to visuo-motor interferences resulting from technological failure of the prosthetic device or interruption of sensorimotor updating after stopping to actively use an attached prosthesis (Castro et al., 2023; Graczyk et al., 2018; Middleton & Ortiz-Catalan, 2020). It is currently unknown whether and how embodiment of a prosthesis can be maintained over longer periods, which would be essential for future clinical applications. Some evidence suggests that prosthesis embodiment might alleviate phantom limb sensations (Bekrater-Bodmann et al., 2021; Lenggenhager et al., 2014; Page et al., 2018; Rognini et al., 2019) and increase compliance with regular prosthesis use (Castro et al., 2023; Engdahl et al., 2020; Graczyk et al., 2018; Murray, 2004). However, experiencing disembodiment of a previously embodied prosthetic hand might work against such beneficial effects of prosthesis-embodiment which is why more research on the question of how embodiment can be maintained over time might be helpful. Along these lines, the current study investigated how experiencing occasional interferences between visual and motor signals or complete interruptions of visuo-motor stimulation during the induction phase of the moving rubber hand illusion impact the temporal dynamics of embodiment and disembodiment of a rubber hand.

### Inducing embodiment: underlying mechanisms

Several studies have investigated how experience of temporal or spatial interferences between tactile or motor signals coming from one’s own hand and visual signals coming from an artificial hand affect the induction of the rubber hand illusion (e.g., Caspar et al, 2015; Riemer et al., 2014; Shimada et al., 2009). For example, one study, compared the synchronous condition of the active and passive version of the rubber hand illusion, where cross-modal stimulation is temporally and spatially correlated between the real and the rubber hand, to an asynchronous condition, where temporal synchrony was violated while spatial compatibility remained intact and to an incongruent condition where the spatial compatibility was violated while temporal synchrony remained intact (Riemer et al., 2014). In the asynchronous condition, temporal correlation between cross-modal signals was disrupted by introducing a temporal delay between signals coming from the real hand and the rubber hand. In the incongruent condition, spatial incompatibility resulted from an alternated mapping of stimulated fingers between the real and the rubber hand, e.g., moving the index finger triggered movements of the rubber hand’s middle finger and vice versa. Sense of embodiment for the rubber hand did not differ between the asynchronous and the incongruent condition and was significantly lower than in the synchronous condition. These findings show that the emergence of the rubber hand illusion follows temporal and spatial rules of bottom-up driven multisensory integration that were also found for other perceptual processes, especially in situations when encoding of each individual signal is impaired due to the signal’s relatively low reliability (Abdulkarim et al., 2021; Ehrsson et al., 2005; Holmes and Spence, 2005; Stein and Stanford, 2008). However, interferences between cross-modal signals during the rubber hand illusion might also conflict with prior knowledge representing previously acquired associations between specific body-related visual signals (e.g., seeing a hand being touched by a brush or seeing one’s own index finger tapping) and tactile, motor, and proprioceptive sensations that usually correlated with the visual signals in the past (Liesner et al., 2021; Verschoor & Hommel, 2017). Furthermore, even if cross-modal signals are temporally synchronous and spatially compatible, inconsistencies with prior knowledge about how one’s own body looks and feels like might still impair multisensory integration (Kalckert et al., 2019; Lin & Jörg, 2016; Petkova & Ehrsson, 2008; Tsakiris et al., 2010; Ward et al., 2015). Causal inference models of multisensory integration account for the joint contribution of bottom-up and top-down mechanisms to bodily self-perception. Such models suggest that multisensory integration occurs if incoming cross-modal signals are perceived as originating from a common source which is more likely when signals are temporally correlated, spatially compatible, and consistent with prior body-related experience (Chancel et al., 2022; Samad et al. 2015).

Thus, it appears that the emergence of the moving rubber hand illusion critically depends on experiencing that real hand movements continuously trigger temporally correlated and spatially compatible movements of the rubber hand. Further, sense of embodiment for the rubber hand increases gradually over the stimulation period and does not pop up instantly after stimulation onset (Abdulkarim et al., 2021; Kalckert & Ehrsson, 2017; Perepelkina et al., 2018; Pfister et al., 2021; Tsakiris & Haggard, 2005). It is interesting that such an induction procedure reveals parallels to operant conditioning, where the probability for occurrence of a behavior, e.g., pressing a lever, depends on whether the behaviour is followed by an effect, e.g., receiving a food pellet (Murphy & Lupfer, 2014). Under this perspective, movements of the rubber index finger would correspond to a reinforcer, while the procedure of coupling of the reinforcer – i.e., the rubber finger movements – with the real finger movements during the induction phase of the moving rubber hand illusion would correspond to a continuous reinforcement schedule because each finger movement is continuously followed by a corresponding movement of the rubber finger. Drawing the analogy with reinforcement schedules is particular interesting for the present research because of the so-called partial reinforcement extinction effect. This effect describes the finding that compared to continuous reinforcement, after partial reinforcement – when not all occurrences of a behavior were reinforced but only some of them – the learned behavior is more resistant to extinction (e.g., Harris, 2024; Pittenger et al., 1988; Rescorla, 1999; Segers et al., 2018; Thrailkill, 2023). For the moving rubber hand illusion this finding might imply that occasional omissions of rubber hand movements while continuing moving the real hand might lead to more stable embodiment of the rubber hand as compared to the standard procedure where each real hand movement is followed by a rubber hand movement.

### The present study

Taken together, evidence from the rubber hand paradigm suggests that embodiment of the rubber hand emerges from continuous experience of correlated multisensory stimulation of one’s own hand and the rubber hand, while continuously experiencing interferences between cross-modal signals prevents emergence of the illusion (Caspar et al., 2015; Riemer et al., 2014; Riemer et al., 2019). However, it is not clear how occasionally experiencing interference might affect embodiment and subsequent disembodiment of an artificial hand. Inspired by findings on the partial reinforcement extinction effect, we hypothesized that occasional instances of interference-experience within a predominantly compatible induction phase might not only not impair embodiment but even result in more stable embodiment (reflected in slower disembodiment) compared to embodiment that emerged from continuous experience of temporally and spatially matching cross-modal signals (Harris, 2024; Segers et al., 2018; Thrailkill, 2023).

We tested the latter assumption in two experiments. The procedure for the reported experiments is based on a previous study on disembodiment (Pfister et al., 2021). The general idea of this study was to induce the moving rubber hand illusion first which then enables to study disembodiment afterwards. Each trial of the experiment thus consisted of two parts, an “embodiment phase” to evoke embodiment of the rubber hand and a subsequent “disembodiment phase” to probe for the temporal dynamics of the embodiment experience. Increase and decrease of embodiment for the rubber hand was measured by means of subjective embodiment ratings that participants provided several times during each phase. The critical manipulation of the present experiment was implemented during the embodiment phase. Participants moved their index finger continuously up and down and most of the movements were instantly followed by corresponding movements of the rubber index finger. However, four times the rubber finger remained still for several seconds while participants continued moving (intermittent condition). In the control condition, congruency between index and rubber finger movements was maintained during the whole embodiment phase (continuous condition). For the continuous condition we predicted a gradual increase of ratings during the embodiment phase and a gradual decrease of ratings during the disembodiment phase (Abdulkarim et al., 2021; Pfister et al., 2021). In line with the partial reinforcement extinction effect, we expected for the intermittent condition a slower increase of ratings for the embodiment phase and a slower decrease of ratings for the disembodiment phase of the intermittent condition compared to the respective phase of the continuous condition (e.g., Hulsbosch et al., 2023; Segers et al., 2018; Thrailkill, 2023).

## Experiment 1

In Experiment 1 we investigated whether occasional interruptions of the contingency between finger movements of the participants and movements of the rubber index finger during the induction phase of a moving rubber hand illusion would result in prolonged and more stable embodiment of the rubber hand.

### Methods

#### Participants

Effects of disembodiment in similar paradigms have previously been observed with an effect size of *d_z_* = 0.78 for the comparison between the embodiment rating right after the active induction phase of the illusion and the embodiment rating after two minutes of passively watching the rubber hand (Pfister et al., 2021). Based on this result a power analysis suggests a sample size of 33 participants to ensure high power of 1–β > 99%. However, we expected that the effect size for the key comparison in the present study might be lower than this previously reported effect. We, therefore, based our calculations on a generic medium effect size of *d_z_* = 0.5 (using the power.t.test function in R 4.3.1). A sample size of 56 participants provided a power of 1–β > 95% for effects of this size. Further, based on previous findings we expected that about 10%–40% of individuals would not respond to the illusion (e.g., Kalckert et al., 2019; Kalckert & Ehrsson, 2014a; Kalckert & Ehrsson, 2014b). The chosen sample size of 56 participants would ensure an acceptable power of 1–β = 89% even in the case of dropouts (assuming a dropout rate of 25%). Note, due to an error, data were collected from 57 instead of the preregistered 56 participants (preregistration for Experiment 1: https://aspredicted.org/tf9xu.pdf). Participants were recruited through the online platform SONA (https://psywue.sona-systems.com) and paid for participation. The experiment was conducted in a laboratory room of our department and informed consent was obtained from each participant. All reported experiments were conducted in accordance with the ethical regulations of the Ethics Committee of the Institute of Psychology, University of Würzburg.

After data processing (see section *Data processing* for more details) the final sample comprised 45 participants for whom 8.1% of trials were excluded. This sample size suggests a drop-out rate of 21% which is within the range of previously reported dropout-rates (Kalckert et al., 2019; Kalckert & Ehrsson, 2014a; Kalckert & Ehrsson, 2014b) and slightly lower than the 25% which we expected for the current study. Participants were on average 29.16 years old (range: 21–66). Most of the participants, 34, were female (seven male, one non-binary, and three did not report their gender). While 37 participants indicated the right hand as their dominant hand, five reported to be left-handed, and three did not report their handedness.

Beyond demographics participants were asked to describe their experience of the mismatch between their own movements and movements of the rubber finger and to share their assumptions about the main research question of the current study if they had any (debriefing questions; Forster et al., 2022). Nineteen participants reported that they have felt irritated, disturbed, or even frustrated during the brief incidents when their movements did not affect the rubber hand. Ten participants attributed the non-reactiveness of the rubber finger to technical issues or erroneous performance and two participants indicated that they did not notice any interruption at all. Although nine participants assumed that the study might be about how synchronization between one’s own movements and movements of the rubber hand affect the body representation, no one guessed the exact research question of the current study – i.e., whether experiencing a mismatch of the contingency between actions and effects during the induction phase result in slower dissolving of the illusion after bringing it about. Therefore, we did not exclude these participants from data analysis.

#### Apparatus & stimuli

**Figure 1:**
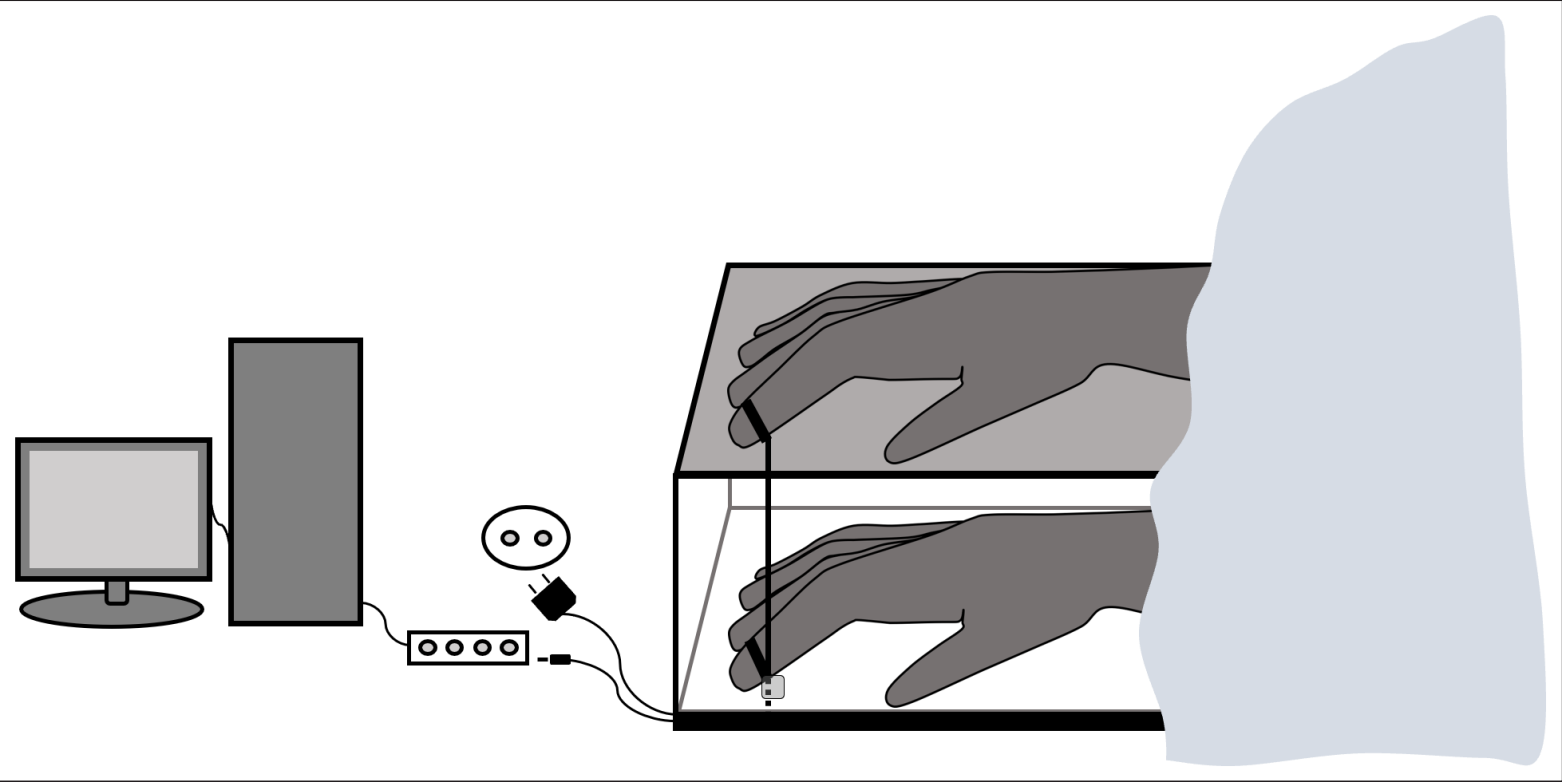
Illustration of the apparatus.

Figure 1 shows the apparatus that we used for the experiment. During the experiment, participants were sitting at a table with a right rubber hand in front of them. The rubber hand was lying on top of a box that was placed near to the participants’ body in an anatomically plausible position. The rubber hand was covered in a disposable glove and its index finger was put into a ring. Before participants inserted their right hand into the box, they had to put on a disposable glove like the one that was on the rubber hand. Participants could not see their right hand when it was in the box. While the position of the rubber hand corresponded to the position of the participants’ hand inside the box in the horizontal plane, in the vertical plane both hands were approximately 10 cm apart. The chosen distance was based on a study investigating how various distances between the artificial and the real hand affect the emergence of the moving rubber hand illusion (Kalckert & Ehrsson, 2014b).

Inside the box participants had to put the tip of the index finger right under a metal device that imitated the ring that participants saw at the rubber index finger. A piece of fabric was put above the end of the rubber arm and the participants arm and shoulder. The two rings inside and outside the box were connected through a thin aluminum tube. A magnet was attached to the ring inside the box and covered under the upper part of the double bottom of the box where it could be connected to electricity supply. The aluminum tube went through the magnet and an iron component was installed inside the tube at the height of the magnet. If electricity was on, the iron part inside the aluminum tube was attracted by the magnet and both, the magnet and the aluminum tube, moved up and down when participants lifted or lowered the ring that was inside the box with the tip of their index finger. This entailed that tapping movements of the real finger inside the box caused corresponding movements of the rubber finger on top of the box. Without electricity supply the aluminum tube was no longer attracted by the magnet and remained still although participants continued moving the ring with the magnet. In this case, finger movements did not affect the rubber hand. The apparatus was connected via a parallel port to the computer and the inflow of electricity could be controlled through a computer program that was written in PsychoPy (Version: 2022.2.4).

#### Procedure

After filling in informed consent at the computer participants received instructions for the experiment in written form. In addition, the procedure was also explained by the experimenter. Then participants were directed to another table where the rubber hand apparatus was placed in front of them. The apparatus was covered with a piece of fabric so that participants could not see it beforehand. Participants were asked to remove jewelry if they had any on their right hand and to put on a disposable glove. Then the experimenter told the participants to close the eyes and inserted the participants’ right hand inside the box. The position of the hand inside the box was adjusted so that the tip of the index finger was right under the ring-like metal device that could be connected to the ring that was on the rubber index finger. The end of the rubber arm as well as the participants’ arm and shoulder were covered with the piece of fabric that was covering the apparatus before. Participants were told to relax their left hand and to put it down on their lap. The experimenter sat at the table with the computer during the experiment and supervised the procedure.

Directly at the start of the experiment participants were asked to submit the first embodiment rating. Subsequent ratings were requested every 30 seconds. The rating procedure entailed that participants evaluate on a scale from 0 to 10 in steps of one to which extend they experience the rubber hand as a part of their body. We provided the following semantic anchors for the rating scale: 0: *I feel no relation between myself and the hand*; 3: *I could imagine that the hand belongs to me;* 7: *I have the feeling that the hand is part of my body;* 10: *I have the feeling that the hand is my own hand*. Participants received a detailed description of the rating question and the rating anchors during instructions before the experiment. During the experiment, participants were verbally prompted by the experimenter whenever submission of a rating was required (“Please submit a rating now.”). To submit a rating, participants responded to the experimenter with a number from the rating scale that best represented their momentary sense of embodiment. The experimenter then entered the rating in the computer program. After participants submitted the first rating the experimenter started to play rhythmic music via the computer. Then, participants were told to perform continuous tapping movements to the beat of the music (85 BPM) with the right index finger. In general, movements of the real index finger caused corresponding movements of the rubber index finger. After two minutes (embodiment phase), right before the submission of the 5^th^ rating, participants were instructed to stop moving and to passively watch the rubber hand for another two minutes (disembodiment phase).

**Figure 2:**
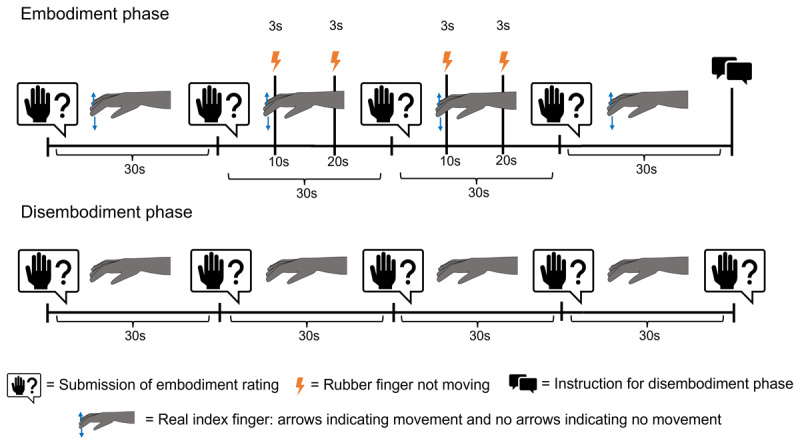
Timeline of events for one trial of the intermittent condition in Experiment 1. Note: The figure illustrates events during one trial of the intermittent condition. Corresponding visual events on the rubber hand were congruent with actions of the real hand most of the time. Interruptions of contingency between action and effect are indicated by the flash icon. The intermittent and continuous condition only differed with respect to these interruptions: while present in the intermittent condition, they were absent in the continuous condition.

While the procedure during the disembodiment phase was the same in both conditions it differed during the embodiment phase. In the continuous condition the index finger inside the box and the rubber index finger on top of the box were constantly connected. As a consequence, there was a perfect match between their movements and movements of the rubber index finger during the embodiment phase of the continuous condition. However, during the embodiment phase of the intermittent condition the real and the rubber index finger were disconnected twice during the second inter-rating interval (2^nd^–3^rd^) and twice during the third inter-rating interval (3^rd^–4^th^ rating) for the duration of 3 seconds respectively resulting in approximately 4 omitted taping movements. Please see Figure 2 for a detailed illustration of the events during one trial.

We had three trials for each of the two conditions and nine ratings during each trial. All participants went through all conditions and rating positions. Conditions alternated trial-wise and it was randomised whether participants started the experiment with the continuous or the intermittent condition. After the last rating of a trial, participants had a break of 30 seconds before starting the next trial. For the duration of the break everything was set to default: the music was turned off, the participants were told to close their eyes, the participants took their arm out of the box and the experimenter covered the apparatus. Then the participants were told to open their eyes and to move the right hand while looking at it for the following 30 seconds. After completing the last trial, participants moved to the table with the computer to indicate demographic information and answer the debriefing questions. All text was presented in German language. It took participants approximately 60 minutes to complete the study.

#### Data processing

As per our preregistration, we excluded all trials from data analysis if at the end of the embodiment phase (5^th^ rating position) participants reported embodiment ratings lower than 3. Because the current study focused on the temporal dynamics of disembodiment this cut-off criteria was implemented to control for successful embodiment induction in the first place. Malfunctioning of the apparatus or other deviations from the intended procedure also led to trial exclusion. Entire datasets were excluded if at the end of the embodiment phase embodiment ratings lower than three were reported twice or more because repeatedly failed embodiment induction is indicative for non-responders (Kalckert et al., 2019; Kalckert and Ehrsson, 2014a, 2014b). Datasets were also excluded completely if filtering of the data resulted in at least one condition without remaining trials.^1^ If not stated otherwise, data analyses were performed as preregistered (https://aspredicted.org/tf9xu.pdf). Any deviations from the preregistration are clearly highlighted as non-preregistered and exploratory.

### Results

**Figure 3:**
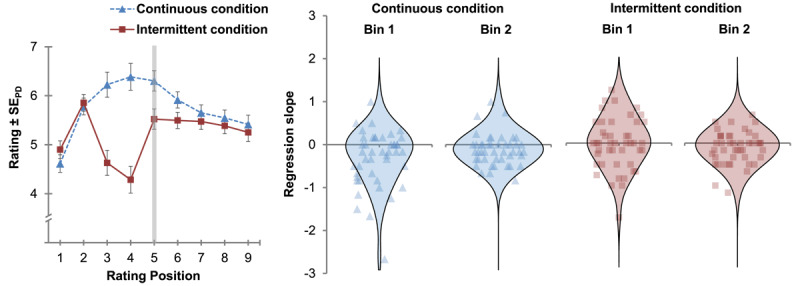
Left panel: Mean embodiment ratings for embodiment and disembodiment phase of Experiment 1 as a function of rating position and condition. Right panel: Violin plots of the distribution of individual regression slopes across participants for the first and second time bin of the disembodiment phase for each condition. Note: The vertical grey line at the 5^th^ rating position of the left panel represents the transition from the embodiment phase to the disembodiment phase. Error bars indicate standard errors (±1 SE) of paired differences between the continuous and the intermittent condition (Pfister & Janczyk, 2013).

Figure 3 shows at the left panel mean embodiment ratings as a function of rating position (1^st^–9^th^ rating position) and condition (continuous vs. intermittent). At the right panel of Figure 3 individual regression slopes across participants are plotted for the disembodiment phase. All analyses for the current study were performed in R (4.3.1). Whenever performing Bayesian statistics (R package BayesFactor using \[\sqrt 2 \]/2 for r-scale) we considered a Bayes. Factor of BF_01_ > = 3 as statistical evidence in favour of equality of the means between conditions. Whenever relying on the frequentist approach, we considered p-values lower than 0.05 as indicating statistical significance. Data and the script for the analysis are available on the Open Science Framework: https://osf.io/anwyh.

#### Embodiment phase

We determined regression slopes for the ratings during the embodiment phase and performed *t*-tests to contrast the means of the regression slopes between conditions and for each condition against zero respectively. Individual regression slopes were significantly different between conditions, *t*(44) = 6.70, *p* < .001, *d_z_* = 1.00. For the continuous condition, the individual regression slopes were significantly greater than zero, *t*(44) = 5.83, *p* < .001, *d_z_* = 0.87, but not for the intermittent condition, *t*(44) = –0.47, *p* = .679, *d_z_* = –0.07. The outcome of Bayesian *t*-tests comparing embodiment ratings between conditions for the 1^st^ and the 5^th^ rating position respectively, neither supported the null hypothesis of equivalence for the first comparison, BF_01_ = 1.95, nor for the second comparison, BF_01_ = 0.02.

We performed further non-preregistered exploratory analyses to investigate the observed pattern of results in more detail. For this we performed an additional *t*-test to contrast for the intermittent condition the 1^st^ rating with the rating that came right after the two inter-rating intervals where the intervention took place (4^th^ rating position), *t*(44) = 1.93, *p* = .060, *d_z_* = 0.39. Further we extracted individual regression slopes for the first inter-rating interval (1^st^ to 2^nd^ rating) and for the last inter-rating interval (4^th^ to 5^th^ rating) of the embodiment phase of the intermittent condition. The mean slopes were comparable between both inter-rating intervals as was suggested by the results of a Bayesian *t*-test, BF_01_ = 4.10.

#### Disembodiment phase

We divided the disembodiment phase in a first (5^th^ to 7^th^ rating positions) and a second time bin (7^th^ to 9^th^ rating positions) and compared extracted mean individual regression slopes in a 2 × 2 ANOVA with the factors time bin and condition (continuous vs. intermittent). The ANOVA revealed a significant main effect of condition *F*(1, 44) = 7.01, *p* = .011, η_p_^2^ = .14 and a significant interaction of both factors, *F*(1, 44) = 4.95, *p* = .031, η_p_^2^ = .10. The main effect of time bin was not significant, *F*(1, 44) < 1. For the first time bin we further computed *t*-tests to contrast individual regression slopes against zero for each condition respectively. Individual regression slopes were significantly below zero for the continuous condition, *t*(44) = –3.32, *p* = .001, *d_z_* = –0.49., whereas there was no significant effect for the intermittent condition, *t*(44) = –0.26, *p* = .396, *d_z_* = –0.04. An additional non-preregistered, exploratory analysis revealed that mean individual regression slopes were significantly different between conditions for the first time bin, *t*(44) = –3.00, *p* = .004, *d_z_* = –0.45, while there was no significant difference between conditions for the second time bin, *t*(44) = –0.10, *p* = .921, *d_z_* = –0.01.

### Discussion

The results of Experiment 1 showed a sharp decline of embodiment ratings during the embodiment phase of the intermittent condition after experiencing periods of mismatch between real and rubber finger movements. This observation suggests that the absence of the expected effects of one’s own movements not only did not enhance the embodiment of the rubber hand but rather actively contradicted it (Pfister et al., 2021). From the perspective of reinforcement learning, it appears that the occasional contradictory evidence (i.e., rubber finger does not move despite moving the real finger) during the acquisition phase resulted in extinction of associations that were learned hitherto. This observation is not compatible with evidence on partial reinforcement schedule-based learning because this would predict that as with continuous reinforcement schedules also for partial reinforcement schedules learning should continuously increase during the acquisition phase, although somewhat slower than under continuous reinforcement (Hulsbosch et al., 2023; Segers et al., 2018). Hence, the current manipulation might have been too strong for the intended implementation of a partial reinforcement schedule because disruption of the contingency between action and effect not only did not update the current body representation but provided sensorimotor evidence against the illusion through violating the temporal and spatial correspondence between motor and visual signals (Pfister et al., 2021). Based on findings that emphasize the importance of temporal correlation and spatial compatibility between cross-modal signals for embodiment (Caspar et al., 2015; Kalckert and Ehrsson, 2014a; Riemer et al., 2014), it seems plausible that in the context of the moving rubber hand illusion it is not so much the experience of contingency between action and effect itself but rather the experience of temporal and spatial correspondence between motor and visual signals which might act as a reinforcer. However, in the moving rubber hand illusion contingency between action and effect and temporal and spatial compatibility between motor and visual signals usually are tightly intertwined. In Experiment 2, we aimed at disentangling the contribution of both factors.

The strong impact of the manipulation in the embodiment phase also affects the interpretation of findings for the disembodiment phase. It is not clear whether the observed differences between conditions for the disembodiment phase reflect effects of our manipulation or whether the flatter decline during the disembodiment phase of the intermittent as compared to the continuous condition stems from preceding differences at the end of the embodiment phase where ratings were lower in the intermittent as compared to the continuous condition. We, therefore, ruled out the latter explanation in Experiment 2.

## Experiment 2

Experiment 2 was designed to address the two main issues limiting the interpretation of Experiment 1.

First, we investigated the question of whether the sharp decline of embodiment ratings during the embodiment phase of the intermittent condition was due to conflicting sensorimotor signals that were inherent to the experienced interruptions of action-effect contingency. Based on evidence suggesting for the continuous condition that gradual increase of embodiment ratings during the embodiment phase and gradual decrease of ratings during the disembodiment phase was due to either presence or absence of continuous sensorimotor updating of the current body representation (Pfister et al., 2021), a partial reinforcement schedule for inducing the rubber hand illusion possibly might be implemented through occasionally interrupting sensorimotor updating. In contrast to interruptions of action-effect contingency, interruptions of sensorimotor updating are not supposed to entail additional conflicting information. To test these assumptions, in Experiment 2, we added a third condition to the design of Experiment 1: the stop-and-go condition (Eck & Pfister, 2024). During the embodiment phase of the stop-and-go condition participants occasionally stopped moving and the participants’ real finger as well as the rubber finger remained still for several seconds. Then participants continued moving like before the short break. We expected that short interruptions of sensorimotor updating that are free of contradictory information during the embodiment phase of the stop-and-go condition would probably slow down but still reinforce embodiment of the rubber hand without causing significant disembodiment. Besides possibly being more suitable for addressing our research question, the stop-and-go condition further might better reflect the situation of prosthesis users. Drastic mismatches between intended action and actual prosthesis performance which are mirrored by the intermittent condition are likely rare (Salminger et al., 2022). However, stopping to move a prosthesis because prosthesis movement is not required for a current task is a process that is an integral part of everyday life activities. Such daily challenges to embodiment are captured by the stop-and-go condition.

The second issue with Experiment 1 was that the interpretation of the results for the disembodiment phase was complicated because of differences between conditions at the end of the embodiment phase. Findings from intermittent learning suggest that with partial as compared to continuous reinforcement schedules learning might need more time (Hulsbosch et al., 2023; Segers et al., 2018). However, in Experiment 1, the length of the embodiment phase was the same for the continuous and the intermittent condition which might have led to the observed large differences between conditions at the end of the embodiment phase. In Experiment 2 we aimed at eliminating previously observed differences between conditions at the end of the embodiment phase through prolonging the embodiment phase of the condition with partial reinforcement. To this end we used an algorithm which enabled flexible adjustment of the length of the embodiment phase for each participant individually. Participants always went through the condition with the continuous reinforcement schedule first. The embodiment phase of the condition with the partial reinforcement schedule was conducted until participants reported the same or higher level of embodiment as they have reported previously at the end of the embodiment phase of the condition with continuous reinforcement (see Methods section below for a detailed description of the algorithm).

We hypothesised that the embodiment phase in the stop-and-go and the intermittent conditions would be longer than in the continuous condition. This would be reflected in a lower number of ratings in the continuous condition relative to the other two conditions, because embodiment ratings were collected every 30 seconds and therefore the number of submitted ratings increases for extended durations of the embodiment phase (see Method section below for more detailed explanation of the procedure). The difference in number of ratings should be smaller between the continuous and the stop-and-go condition than between the continuous and the intermittent condition. We expect that at the end of the embodiment phase ratings should not differ between conditions, due to the adjustment of the length of the embodiment phase according to the respective reinforcement schedule. Further, if implementation of partial (stop-and-go or intermittent condition) reinforcement schedules during the embodiment phase was successful in terms of a partial reinforcement extinction effect, then, during the disembodiment phase ratings should decrease faster in the continuous than in the other two conditions. This assumption is based on findings from operant conditioning showing more persistent learning with partial than with continuous reinforcement schedules (Harris, 2024; Segers et al., 2018; Thrailkill, 2023).

### Method

#### Participants

We collected data from 60 new and naïve participants. The sample size was based on the power calculations from Experiment 1 which suggested that a sample size of 56 participants would ensure acceptable power (1–β > 95%). However, we increased the sample size for Experiment 2 as much as possible with regard to the available time schedule for data collection to ensure more power because results of Experiment 1 might suggest that we possibly might have overestimated the effect size (*d*_z_ = 0.5) when we estimated the sample size for Experiment 1. After filtering the data for non-valid blocks and datasets (see *Data processing* section below for more details on the data exclusion procedure) the final sample (40 female, 11 male) consisted of 51 participants for whom 5.23% of blocks were excluded. The participants’ mean age was 26.45 years (range: 18–68, two missing values). Eight participants reported left-hand dominance while the remaining participants reported the right hand as the dominant hand.

#### Apparatus, stimuli, & procedure

The apparatus, stimuli, and the general setting were the same as in Experiment 1. Therefore, in the following, we report only the main changes that we implemented in Experiment 2 relative to the design and procedure of Experiment 1 (the pre-registration is available at https://aspredicted.org/blind.php?x=4ZX_LL8).

In the new stop-and-go condition participants were instructed four times during the embodiment phase to stop moving for three seconds and to resume moving thereafter. The timing of these movement-interruptions in the stop-and-go condition was analogous to the timing of the interfering events (i.e., disconnection of the real and rubber finger) during the intermittent condition (see Figure 2). Participants stopped moving when the experimenter said “Stop” (in German: “Stopp”) and continued moving as soon as the experimenter said “Go” (in German: “Los”). We further adjusted the order of the conditions and the length of the embodiment phase for the stop-and-go and the intermittent condition in Experiment 2. The continuous condition always came first and was followed by the other two conditions. The length of the embodiment phase of the intermittent and the stop-and-go condition was prolonged until participants reported the same or higher level of embodiment as they have reported at the end of the embodiment phase of the continuous condition – which would correspond to the 5^th^ rating position because in the continuous condition we had constantly 5 ratings. For example, if in the continuous condition a participant had reported an embodiment rating of 6 at the 5^th^ rating position, and in the following intermittent or the stop-and-go condition the same participant reported a rating of 4 at the 5^th^ rating position than the participant had to continue tapping and observing synchronous movements of the rubber finger for another 30 seconds. After this first extension of the embodiment phase the participant had to submit a further rating which would now correspond to the 6^th^ rating position. If this rating was 6 or higher (in our example 6 was the last rating that had been previously submitted for the embodiment phase of the continuous condition), then the participant would continue with the disembodiment phase. However, if the rating at the 6^th^ rating position was lower than 6, then for this participant the embodiment phase would have been prolonged for another 30 seconds and a further rating (7^th^ rating position) would have been collected thereafter. The overall extension of the embodiment phase was limited to 90 seconds. Therefore, after submitting the 8^th^ rating, participants always continued with the disembodiment phase, regardless of whether the current rating was lower, equally high, or higher than the rating that they had submitted previously at the end of the continuous condition. Thus, while during the embodiment phase of the continuous condition we always collected five ratings, during the embodiment phase of the other two conditions there could be more ratings with a maximum of eight ratings. Because of this adaptive procedure, in the instruction for the disembodiment phase came right after the last rating of the embodiment phase while it was presented right before the last rating of the embodiment phase in Experiment 1.

All participants performed three blocks each consisting of one trial of each of the three conditions (9 trials in total). In each block the continuous condition came always first and was then followed by the intermittent or the stop-and-go condition. The order of the intermittent and the stop-and-go condition was counterbalanced across participants. The overall duration of Experiment 2 was approximately 15 minutes longer than Experiment 1 (approx. 75 min). To not prolong the experiment even further we asked only demographic questions at the end of the experiment and skipped the debriefing questions. In Experiment 1 many participants reported experiencing the occasional interruptions of the connection between one’s own movements and movements of the rubber hand as disturbing. In Experiment 2 we intended to prevent possible occurrence of negative feelings through explicitly telling participants during instructions that occasionally, the rubber finger might not move despite them continuing moving and that sometimes they would be prompted to stop moving.

#### Data processing

Exclusion criteria for Experiment 2 were mostly the same as for Experiment 1 with one important exception: In Experiment 1, we had only screened for non-responders in the continuous condition. However, in Experiment 2, the level of embodiment at the end of the embodiment phase of the continuous condition critically affected ratings and the length of the embodiment phase of the other two conditions. Therefore, if embodiment ratings at the end of the embodiment phase of the continuous condition were lower than 3, we not only excluded trials for this condition but a whole block consisting of one trial for each condition (Note, excluding blocks instead of single trials is a slight deviation to the preregistered procedure for data exclusion). Statistical analyses were conducted as preregistered (https://aspredicted.org/blind.php?x=4ZX_LL8). Any deviation from the preregistration is pointed out as exploratory.

### Results

**Figure 4:**
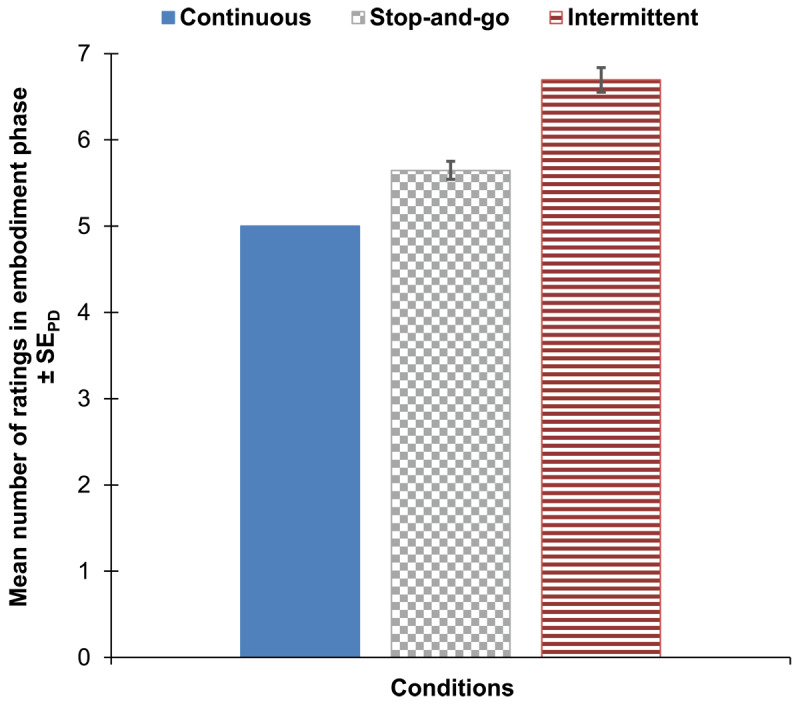
Mean number of ratings in embodiment phase of Experiment 2 for each condition. Note: Ratings were collected every 30 seconds. Therefore, the number of ratings represents the duration of the embodiment phase because with each additional rating the embodiment phase was extended for 30 seconds. For the stop-and-go condition (bar with grey squares pattern), error bars indicate standard errors (±1 SE) of paired differences between the stop-and-go and the continuous condition. For the intermittent condition (bar with red lines pattern), error bars indicate standard errors (±1 SE) of paired differences between the intermittent and the stop-and-go condition (Pfister & Janczyk, 2013).

**Figure 5:**
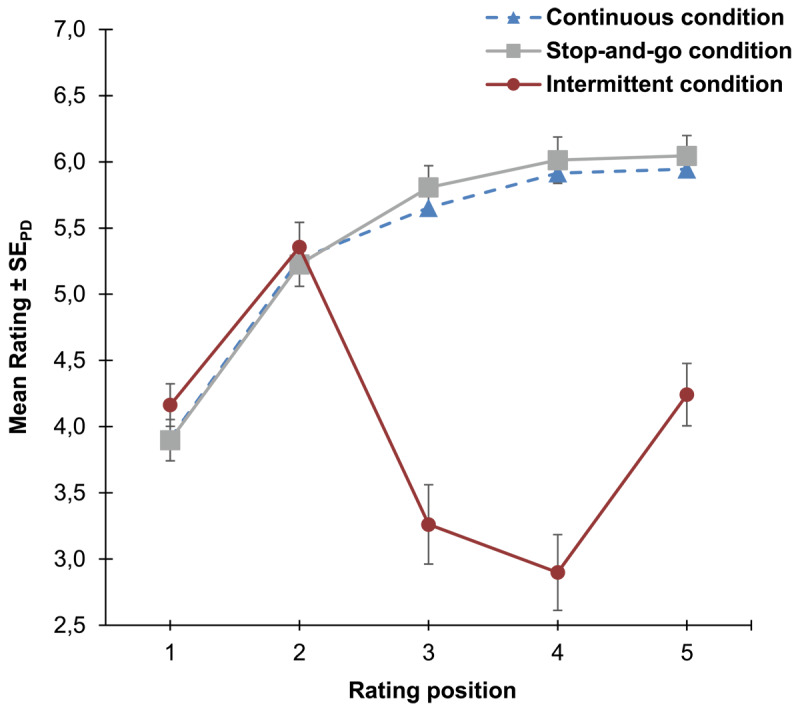
Mean ratings for the first five rating positions of the embodiment phase for each condition of Experiment 2. Note: For the stop-and-go condition (line with grey squares), error bars indicate standard errors (±1 SE) of paired differences between the stop-and-go and the continuous condition. For the intermittent condition (line with red circles), error bars indicate standard errors (±1 SE) of paired differences between the intermittent and the continuous condition (Pfister & Janczyk, 2013).

**Figure 6:**
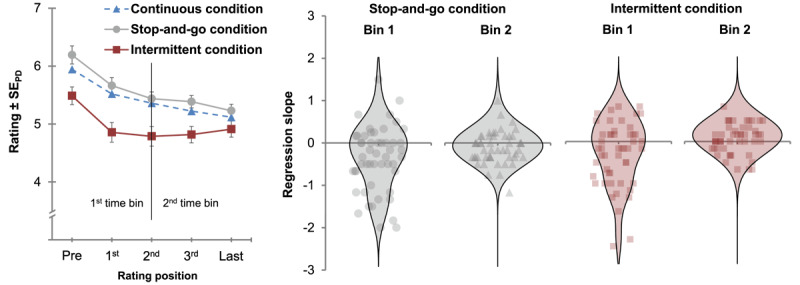
Left panel: Mean embodiment ratings as a function of rating position and condition for the disembodiment phase of Experiment 2 Right panel: Violin plots of the distribution of individual regression slopes across participants for the stop-and-go condition and the intermittent condition. Note: The “Pre”-rating is the last rating of the embodiment phase. For the stop-and-go condition, error bars indicate standard errors (±1 SE) of paired differences between the stop-and-go and the continuous condition. For the intermittent condition, error bars indicate standard errors (±1 SE) of paired differences between the intermittent and the continuous condition (Pfister & Janczyk, 2013).

Figure 4 depicts the mean number of ratings for each condition which represents the amount of extension for the embodiment phase of the stop-and-go and the intermittent condition relative to the embodiment phase of the continuous condition. The mean embodiment ratings for the first five rating positions of the embodiment phase are illustrated in Figure 5 for each condition separately. In Figure 6 the mean embodiment ratings are plotted for the disembodiment phase as a function of rating position and condition (left panel). The right panel of Figure 6 illustrates the distribution of individual regression slopes across participants for the stop-and-go condition and the intermittent condition.

#### Embodiment phase

We compared the mean number of ratings provided (i.e., repetitions until the criterion was reached) between the three conditions in a repeated-measures ANOVA. The main effect of condition was significant, *F*(2, 100) = 92.40, *p* < .001, η_p_^2^ = .65. We further computed t-tests to determine the pattern of the main effect. The contrast between the continuous and the stop-and-go condition was significant, *t*(50) = 6.25, *p* < .001, *d_z_* = 0.87, as was the contrast between the stop-and-go and the intermittent condition, *t*(50) = 7.41, *p* < .001, *d_z_* = 1.04. This suggests that the highest number of rating repetitions was observed in the intermittent condition and the lowest in the continuous condition (see Figure 4). Further the result of an additional non-preregistered and exploratory t-test revealed that the difference between the stop-and-go and the continuous condition was significantly smaller than the difference between the intermittent and the stop-and-go condition, *t*(50) = 7.41, *p* < .001, *d_z_* = 1.04.

In a pre-registered explorative secondary analysis, we further tested whether ratings at the beginning of the embodiment phase differed between conditions. Ratings were comparably high between the continuous and the stop-and-go condition, as the result of a Bayesian t-test suggested, BF_01_ = 6.54. Initial ratings in the intermittent condition were significantly higher than in the stop-and-go condition, *t*(50) = 2.63, *p* = .011, *d_z_* = 0.37, but were not significantly different from the first ratings in the continuous condition. A Bayesian t-test comparing first ratings between the intermittent and the continuous condition did not support the null hypothesis of equality for this comparison, BF_01_ = 2.11. In an exploratory analysis, we further compared ratings at the end of the embodiment phase between conditions. Ratings were significantly lower in the intermittent condition relative to the stop-and-go, *t*(50) = 3.63, *p* = .001, *d_z_* = 0.51, and the continuous condition, *t*(50) = 3.00, *p* = .004, *d_z_* = 0.42. Ratings were not significantly different between the continuous and the stop-and-go condition, *t*(50) = 1.60, *p* = .117, *d_z_* = 0.22, though the null hypothesis of equality was not supported by the result of a Bayesian *t*-test, BF_01_ = 2.01. We did not perform the other part of the preregistered exploratory secondary analysis where we intended to perform Bayesian t-tests to compare ratings before and after the intervention as well as ratings after the intervention with the initial ratings for the intermittent and the stop-and-go condition respectively, because facing the actual data, we realized that these exploratory tests would not add any valuable information.

#### Disembodiment phase

After extracting individual regression slopes for the three conditions, we performed 2 × 3 ANOVA with the factors time bin (1^th^ time bin: last rating of embodiment phase–second rating of disembodiment phase; 2^nd^ time bin: second rating of disembodiment phase–last rating of disembodiment phase). The ANOVA revealed a significant main effect of time bin, *F*(1, 50) = 11.62, *p* = .001, η_p_^2^ = .19, and a non-significant trend for the main effect of condition, *F*(2, 100) = 2.48, *p* = .089, η_p_^2^ = .05. The interaction between both factors was not significant, *F*(2, 100) = 2.36, *p* = .115, η_p_^2^ = .05. Results of t-tests suggested that during the first time bin individual regression slopes were negative in all conditions (continuous: *t*(50) = –2.59, *p* = .006, *d_z_* = –0.36; stop-and-go: *t*(50) = –3.54, *p* < .001, *d_z_* = –0.50; intermittent: *t*(50) = –3.09, *p* = .002, *d_z_* = –0.43).

### Discussion

In Experiment 2 we replicated the sharp decline of ratings during the embodiment phase of the intermittent condition that we have previously observed in Experiment 1. Together, this robust pattern of results suggests that even short experiences of congruency violations between one’s own actions (i.e., movements the real index finger inside the box) and the corresponding action effect (i.e., no movements of the rubber index finger on top of the box) not only do not further reinforce embodiment of the rubber hand but rather breaks the illusion. How sustainable the effect of the interference-experience was on the embodiment experience is further reflected in the number of ratings and the value of the last rating of the embodiment phase. Irrespective of the embodiment phase duration, which was greater for the intermittent condition than for the other two conditions, at the end of the embodiment phase ratings for the intermittent condition were the lowest.

In the stop-and-go condition participants, as predicted, needed less time than in the intermittent condition but slightly more time than in the continuous condition to reach a comparably high or higher level of embodiment compared to the level of embodiment at the end of the continuous condition. Ratings at the end of the embodiment phase were slightly higher in the stop-and-go condition than in the continuous condition. However, this difference was not significant. Altogether, results for the embodiment phase of the stop-and-go condition relative to the continuous condition suggest that the occasional interruptions of sensorimotor updating that participants experienced in the stop-and-go condition did not impair the induction of the rubber hand illusion. Like in the continuous condition, ratings in the stop-and-go condition continuously increased despite these interruptions, although slightly slower than in the continuous condition.

While the results for the embodiment phase of the stop-and-go condition suggest that partial reinforcement might work with the rubber hand illusion, there is no indication for a partial reinforcement extinction effect during the disembodiment phase. In the continuous and the stop-and-go condition ratings gradually decreased during the whole disembodiment phase with most of the decrease evolving during the first part of the disembodiment phase. In the intermittent condition, possibly due to the relatively low level of embodiment at the end of the embodiment phase, ratings only decreased during the first part of the disembodiment phase and remained stable thereafter. Taken together, disembodiment after stopping to move could neither be postponed or slowed down by experiencing action-effect contingency violations nor by the occasional experience of interruptions of sensorimotor updating during the induction phase.

A somehow puzzling observation is that at the beginning of the embodiment phase ratings in the intermittent condition were higher than in the stop-and-go condition. Due to data processing and filtering, the stop-and-go condition was preceded in 24 blocks by the continuous condition and in 27 blocks by the intermittent condition. Re-embodiment after the intermittent condition might have been more difficult than after the continuous condition because in the intermittent condition overall embodiment of the rubber hand was lower and disembodiment of the rubber hand occurred earlier in time and therefore lasted longer, than in the continuous condition. However, it is unlikely that differences between conditions at the beginning of the embodiment phase did affect the overall pattern of the main findings for the embodiment and the disembodiment phase.

## General Discussion

Substantial evidence suggests that continuous experience of incongruent multisensory stimulation or of interruption of sensorimotor updating counters the rubber hand illusion (Caspar et al., 2015; Riemer et al., 2014; Pfister et al., 2021). Against this background, the current study tested how such interferences affect embodiment when they occur only sporadically. For this we compared the dynamics of embodiment and disembodiment for three different embodiment-induction procedures. In the continuous condition we used the standard rubber hand illusion procedure, where each real hand movement triggers a corresponding movement of the rubber hand. In the intermittent condition, the correspondence between real and rubber hand movements was disrupted occasionally (no rubber hand movements despite ongoing real hand movements). In the stop-and-go condition, incoming correlated multisensory signals necessary for updating of the body representation were interrupted intermittently (movement pauses for both hands). The design of the experiments was inspired by operant conditioning paradigms where the continuous condition corresponds to continuous reinforcement schedules while the other two conditions provide two different implementations of partial reinforcement schedules. Findings on the partial reinforcement extinction effect suggest that continuously reinforced behavior is easier to learn but also less resistant to extinction compared to behavior that was partially reinforced during the acquisition phase (Harris, 2024; Hulsbosch et al., 2023; Segers et al., 2018; Thrailkill, 2023). Based on this, we hypothesized for the intermittent (Experiment 1) and the stop-and-go condition (Experiment 2) that relative to the continuous condition, embodiment would emerge more slowly during the induction phase and also dissolve more slowly thereafter, indicated by slower increase and slower decrease of embodiment ratings. For the intermittent condition, we observed that after a brief experience of interference between real and rubber hand movements embodiment ratings rapidly decreased which substantially impaired embodiment induction. This pattern complicated any further interpretation of differences between conditions regarding disembodiment. For the stop-and-go condition, a markedly slower increase of embodiment ratings relative to the continuous condition did support the first part of our hypothesis. However, the observed comparable dynamics of disembodiment between these two conditions is incompatible with the second part of our hypothesis. Overall, we did not find evidence for a data pattern that would follow the partial reinforcement extinction effect. Although methodical overlaps between a typical operant conditioning paradigm and the moving rubber hand illusion paradigm motivated the current study, highlighting the differences might possibly explain the missing support for the partial reinforcement extinction effect in the current data. While operant conditioning is about the acquisition of specific goal-directed behavior through reinforcement the moving rubber hand illusion aims at modifying the body representation through induction of coupled sensorimotor experiences. Although associative learning mechanisms might be involved in both processes, the content of the formed associations diverge (e.g., Kalckert & Ehrsson, 2014a; Rescorla, 1987). Possibly because of such differences effects found in one paradigm cannot be easily transferred to the other.

Results for the continuous and the intermittent condition, which were observed across both reported experiments, replicate findings from an earlier study that investigated the disembodiment of a previously embodied rubber hand (Pfister et al., 2021). In this study, a moving rubber hand illusion was induced first, and then disembodiment of the previously embodied rubber hand was prompted by comparing three conditions. In a control condition the procedure during the disembodiment phase was the same as during the preceding embodiment phase. For the disembodiment phase of the no-movement condition, participants stopped moving and passively observed the passive rubber hand. For the disembodiment phase of the disruption-condition the rubber hand was hit with a hammer right after the end of the embodiment phase. Ratings remained stable only in the control condition. By contrast, embodiment ratings gradually decreased over time in the no-movement condition, and they dropped instantly in the disruption condition. Obviously, the procedure and results for the continuous condition of the current study mirror the procedure and results for the no-movement condition of the Pfister et al. (2021) study. Findings from both studies suggest that continuous experience of synchronous multisensory stimulation is not only necessary for induction but also for maintenance of a sense of embodiment for an artificial limb. When sensorimotor updating stops, then previously established bindings between cross-modal signals gradually dissolve.

However, such gradual multisensory disintegration can be expedited through sensorimotor information that contradicts the current state of embodiment as is suggested by the instant disembodiment that was observed for the intermittent condition in the current study and also for the disruption condition in the Pfister et al. (2021) study. Although the procedures differ between these two conditions, there are important parallels that might explain the similar results. In both studies, instant disembodiment was observed after implementing an interference between signals coming from the rubber hand and signals coming from the real hand. While in the Pfister et al. (2021) study the visual perception of a hand that is being hit was incompatible with absence of tactile perception of the hammer on the skin and absence of pain, in the current study, the visual perception of a passive hand was incompatible with motor perception of finger movements (Gentile et al., 2013; Newport & Gilpin, 2011). In both studies, the experienced interference might have partly resulted from disruption of spatial and temporal correspondence between signals coming from the real and the rubber hand. However, also inconsistency with body-related knowledge might have worked against the illusion as prior experience would suggest that a hand that is being hit but does not hurt as well as a hand that feels active but looks passive is probably not one’s own hand (Liesner et al., 2021; Verschoor & Hommel, 2017).

According to causal inference models, multisensory integration of currently perceived cross-modal signals depends on whether a common cause is inferred for the current sensorimotor experience. The better the temporal and spatial match between the currently perceived signals and the more consistent the current sensorimotor experience is with prior knowledge the more likely is a common cause inferred (Chancel et al., 2022; Samad et al., 2015). The current observation of a gradual increase of embodiment ratings during illusion induction and subsequent gradual decrease for the continuous condition possibly reflects a gradual transition from accumulating evidence for a common cause to accumulating evidence against a common cause for the perceived cross-modal signals. However, the observed rapid decrease of embodiment ratings for the embodiment phase of the intermittent condition, indicates that the induced incongruency experience provided strong evidence against the assumption of a common cause which broke the hitherto emerged embodiment experience. Therefore, possibly, implementation of a more subtle mismatch, like reduced correspondence between the movement amplitude of real and rubber finger or a small delay for the rubber finger movement, might be more suitable for investigating of the hypothesised effects. These manipulations can be expected to conflict less strongly with rubber hand embodiment than the current mismatch experience. Future studies could investigate the suggested transition from sensorimotor evidence for or against the rubber hand illusion in more detail. In particular, it would be interesting to test to which degree interference experience is still compatible with the rubber hand illusion and what sort of counterevidence results in gradual decrease of the illusion or instantly abolishes it (Knoblich & Kirchner, 2004; Riemer et al., 2019; Shimada et al., 2009; Shimada et al., 2014).

Whereas the comparison of the intermittent and the continuous condition did not support our hypotheses, there was some support for partial reinforcement effects in the rubber hand illusion for the embodiment phase of the stop-and-go condition. The observed continuous increase of embodiment ratings during the embodiment phase of the stop-and-go condition was slightly slower relative to the continuous condition, in line with findings showing slower acquisition with intermittent reinforcement schedules compared to continuous reinforcement schedules (Hulsbosch et al., 2023; Segers et al., 2018). Nevertheless, the most important aspect of the partial reinforcement extinction effect is the higher resistance to extinction of behavior that was learned with partial reinforcement as compared to continuous reinforcement. In contrast to our hypothesis of a slower decrease for the stop-and-go condition relative to the continuous condition, we observed comparable temporal dynamics of disembodiment for both conditions. Therefore, it appears as if the results of the stop-and-go condition can be better explained by multisensory integration accounts than by operant learning mechanisms as well (e.g., Gentile et al., 2013; Newport & Gilpin, 2011).

The observed longer duration of the embodiment phase in the stop-and-go condition relative to the continuous condition possibly reflects that the number of experienced instances of multisensory stimulation is positively related to the strength of embodiment (Kalckert, 2018; Sivasubramaniam et al., 2022). In the current study, participants moved their hidden index finger with a frequency of approximately 1.3 Hz. For the continuous condition this resulted in experiencing nearly 160 instances of correlated visuo-motor stimulation per trial. In the stop-and-go condition, visuo-motor stimulation stopped four times with each interruption lasting about three seconds resulting in omission of approximately 16 instances of visuo-motor stimulation per trial. Therefore, to reach an embodiment level which is comparably high to the level of embodiment at the end of the embodiment phase of the continuous condition, the omitted instances of multisensory stimulation had to be compensated. This explanation is further supported by the descriptive finding that embodiment ratings were slightly higher at the end of the stop-and-go condition as compared to the continuous condition. On average, the embodiment phase during the stop-and-go condition was extended for approximately one inter-rating interval which lasts 30 seconds. In 30 seconds, participants had the opportunity to collect more than the omitted 16 instances of visuo-motor stimulation. This might have resulted in a slightly higher total number of experienced multisensory stimulations in the stop-and-go than in the continuous condition. However, the hypothesis that the number of experienced occurrences of multisensory stimulation determines the strength and temporal evolution of embodiment has not been tested directly yet. Future studies might provide empirical evidence for the current speculations through directly comparing the temporal dynamics of the emergence of embodiment in the moving rubber hand illusion between conditions which differ with respect to the number of occurrences of synchronous multisensory stimulation during the induction phase.

### Implications

The current findings come with several implications for prosthetics devices developers and therapists that supervise prosthesis use (Engdahl et al., 2020; Gouzien et al., 2017; Manz et al., 2022). First, the observation of gradual increase of embodiment ratings during continuous experience of congruency between own active movements and movements of the rubber hand suggests that sense of embodiment for an artificial limb need some time to emerge. This finding fits well with results from studies on prosthesis users’ experiences suggesting a positive relationship between wearing time and perception of the prosthesis as an integrated part of one’s own body rather than an external object (Castro et al., 2023; Engdahl et al., 2020; Manz et al., 2022; Murray, 2004). Together, this evidence implies that prosthetic device users should be encouraged to actively use their prosthesis for some time despite possible intentions to reject it due to initial adaptation problems (Salminger et al., 2022). Second, we found that embodiment ratings gradually decreased over time during a 2 min interruption of sensorimotor updating while short interruptions of sensorimotor updating of several seconds had no effect on the current embodiment experience. These observations suggest that when stopping to actively use a prosthetic device for several minutes, sense of embodiment for the artificial limb that emerged during preceding active use might decrease even if the prosthesis remains attached to the rest of the body. In contrast, occasional, short interruptions of active use neither promote disembodiment nor enhance embodiment. Crucially, the finding of a rapid decrease of ratings after the experience of a mismatch between movements of one’s own hand and movements of the rubber hand suggests that sense of embodiment for a prosthesis might instantly dissolve if it does not move as it is expected to move based on current motor perception and prior experience.

However, it is important to point out that the discussed implications were derived from data that was collected from participants who used their intact index finger to control movements of the rubber index finger. While about 10%–40% of individuals who have intact limbs appear to be resistant to the rubber hand illusion (e.g., Kalckert et al., 2019; Kalckert & Ehrsson, 2014a; Kalckert & Ehrsson, 2014b), within the population of individuals who have lost a limb induction of the rubber hand illusion might fail even more frequently. For example, multisensory integration might be more or less difficult for prosthesis users depending on the level of restoration of motor control and proprioception in the stump after amputation because this might substantially affect prosthetic control and perception of sensorimotor feedback (Song et al., 2022; Srinivasan et al., 2017). In addition, cortical reorganisation that is often observed after amputation might further impair multisensory integration (Castro et al, 2023). Finally, embodiment experience with a prosthetic device might differ with respect to the cause of limb absence. Prosthesis users who have lost their limb later in life acquired specific associations between visual, tactile, proprioceptive, and motor signals that are characteristic for specific limb movements prior to amputation and commonly experience phantom limb sensations post-amputation. Prosthesis users with congenital limb absence had no prior experience with the missing limb and are less likely to experience phantom limb sensations (Engdahl et al., 2020; Liesner et al, 2021; Murray, 2008; Verschoor & Hommel, 2017). However, some evidence suggests, that the quality, presence, or absence of phantom limb sensations might substantially impact embodiment experience with a prosthesis (Ehrsson et al., 2008; Murray, 2008). Thus, multisensory processing might not only differ between individuals with intact limbs and prosthesis users but although greatly vary within the population of prosthesis users. It is therefore pivotal to include actual prosthetic device users in future studies to test the proposed implications from the current data for prosthesis embodiment.

### Conclusions

We did not find any evidence for a partial reinforcement extinction effect in the moving rubber hand illusion. Instead, findings from the current study confirm and extend previous multisensory integration accounts of embodiment and disembodiment. These findings suggest that multisensory integration of visual and motor signals in the moving rubber hand illusion is more likely the better signals match in terms of temporal and spatial correspondence and the more the current sensorimotor experience is consistent with prior body-related knowledge. We further showed that similar processes might underly the disintegration of the previously integrated cross-modal signals. Inversely to embodiment, disembodiment of a previously embodied artificial limb is the more likely the less signals that are coming from the rubber hand and the real hand correspond and the more the current sensorimotor experience contradicts prior knowledge about how one’s own body feels and looks like.

## Data Accessibility Statement

The data, scripts, computer programs and stimulus material as well as a link to the preregistrations for all reported experiments are available online (https://osf.io/anwyh).

## Additional File

The additional file for this article can be found as follows:

10.5334/joc.427.s1 Supplemental Material. Data plots for excluded participants.

## Footnotes

Data plots for excluded participants are provided in the supplemental material. We kindly thank one reviewer of the original version of our manuscript for suggesting this supplement.
