# Supplementary material for: After a Hand Was Lent: Sporadically Experiencing Multisensory Interference During the Rubber Hand Illusion Does Not Shield Against Disembodiment
Source: J Cogn. 2025 Jan 17;8(1):18. doi: 10.5334/joc.427 (PMC11740722; doi:10.5334/joc.427)

Supplemental Material – Data plots for excluded participants

Experiment 1 (*N* = 12)

Mean embodiment ratings as a function of rating position and condition for embodiment and disembodiment phase (gray vertical line marks the transition from one phase to another)

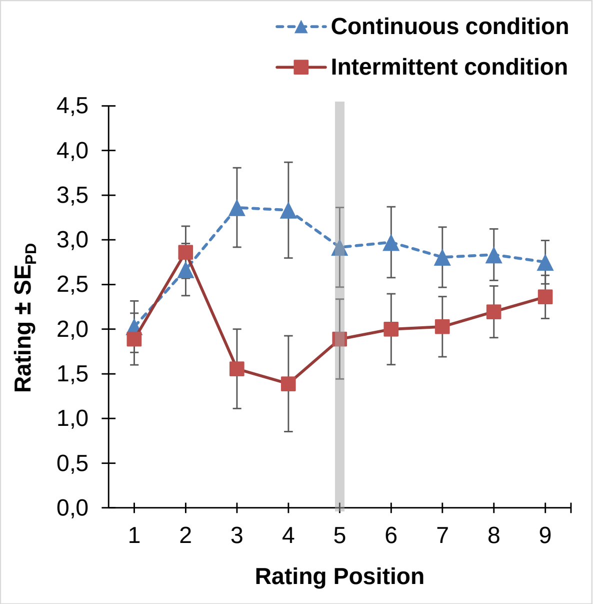

Experiment 2 (*N* = 9)

Mean number of ratings in embodiment phase in each condition

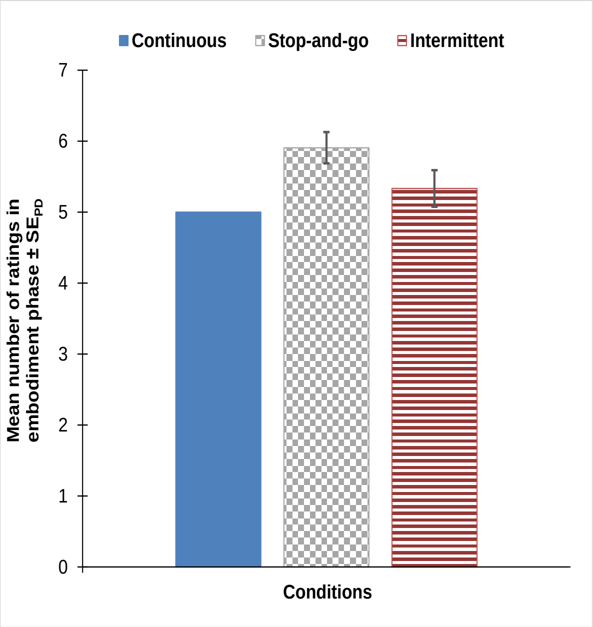

Mean embodiment ratings as a function of rating position and condition for embodiment phase only

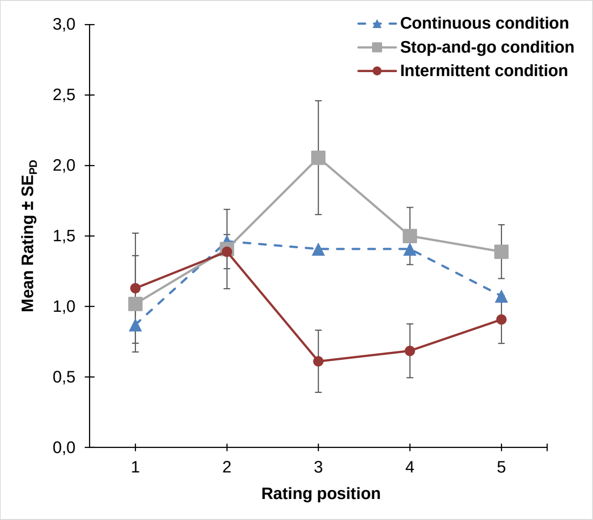

Mean embodiment ratings as a function of rating position and condition for disembodiment phase only

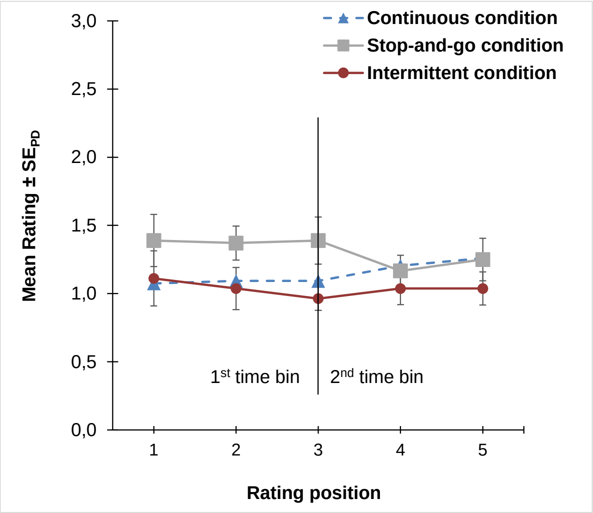

Supplement: Supplemental Material. — Data plots for excluded participants. [file joc-8-1-427-s1.pdf]
